# Spectral proof for the 4-aminophenyl disulfide plasma assisted catalytic reaction

**DOI:** 10.1038/s41598-017-04658-w

**Published:** 2017-06-28

**Authors:** Lixin Xia, Shiwei Wu, Jing Wang, Caiqing Ma, Peng Song

**Affiliations:** 1 Department of Chemistry, Liaoning University, Shenyang, 110036 P. R. China; 2 Department of Physics, Liaoning University, Shenyang, 110036 P. R. China

## Abstract

4-Aminophenyl disulfide (APDS) forms on the surface of silver nanoparticles due to chemical adsorption and disulfide bond breakage. This leads to the formation of new silver chemical bonds to result in the new compound NH_2_-C_6_H_6_-S-Ag. This novel material produces enhanced Raman spectra under weak laser light irradiation. When irradiated a plasma-assisted catalytic coupling reaction of NH_2_-C_6_H_6_-S-Ag occurs leading to the formation of 4,4-dimercaptoazobenzene (DMAB). Raman spectroscopy was used to monitor this reaction process, showing clear spectral changes associated with each step after addition of Ag nanoparticles onto the APDS powder. This method clearly shows the mechanism of the plasma-assisted catalytic reaction and may also be useful for spectral imaging purposes.

## Introduction

Surface enhanced Raman spectroscopy (SERS) is a technique utilized for analysis and testing, and owing to the rapid and simple sample requirements it is employed in a wide range of applications^1–7^. SERS is particularly useful for following reaction processes and confirming reaction products. For example, in 2010, the three so-called b_2_ enhancement peaks expected in the SERS spectrum of 4-aminothiophenol (PATP) were absent, leading to the discovery of a new material, DMAB. This new material forms by a plasma-assisted catalytic coupling reaction under SERS conditions, a process which was later confirmed by experimental and theoretical studies^8, 9^. Subsequent experiments have demonstrated that plasma assisted reactions occur under SERS conditions due to plasma-assisted deposition^10–20^. Importantly, this discovery provided a new method for synthesizing new molecules via a plasma-assisted mechanism, alongside showing SERS as a convenient method for monitoring plasma-assisted catalytic reactions. Furthermore, the use of SERS spectroscopy to monitor plasma-assisted catalytic reactions is also useful for understanding the experimental reaction process and analysing the experimental principles to more completely understand the plasma-assisted catalytic reaction process.

Aromatic disulfide reversible cross-linked polymers are introduced into systems in the field of intelligent materials^21^, for example, APDS can be used as a reusable handle, as a repairable and recyclable epoxy curing agent network^22^, alongside being an important raw material. UV and SERS spectroscopy techniques can be used to monitor the catalytic coupling process of APDS. As changes in the spectra are clearly observed, there action progress can be readily monitored and rationalised. This provides a simple and intuitive method for understanding plasma-assisted catalytic reactions and analysing the experimental principles. Alongside this, we provide a new SERS measurement technology through the addition of silver nanoparticles. This method is simple and fast, with a high repetition rate and is easily imaged. Thus overall, there are potential applications in molecular detection, experimental monitoring and molecular imaging.

## Results and Discussion

**Figure 1:**
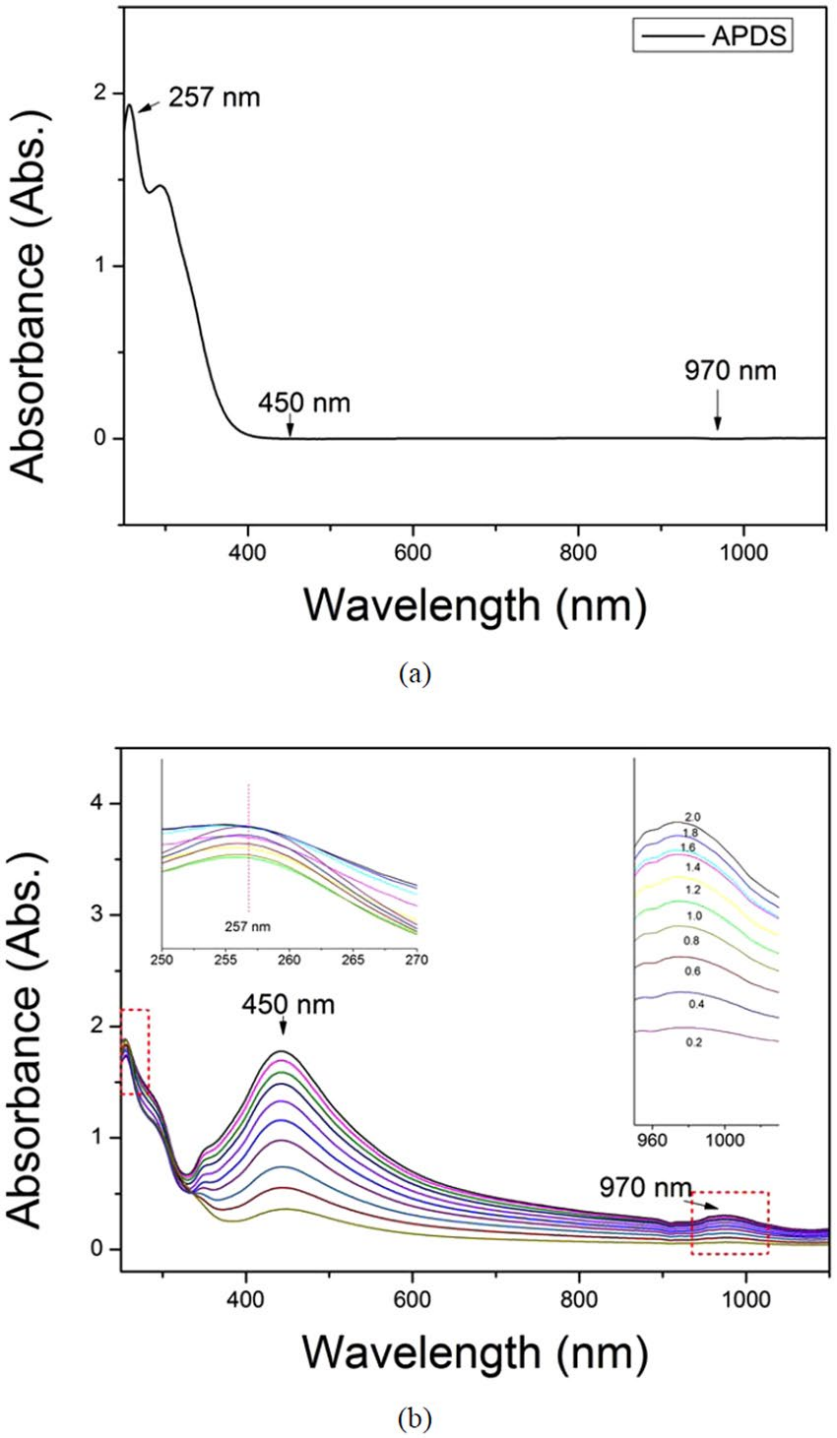
(**a**) The absorption spectra of 10^−5^ M APDS in ethanol solution, (**b**) the spectra mixed with different concentrations of the Ag NPs sol.

The UV-vis spectrum of APDS solution in the absence of Ag sol shows an absorption peak at 257 nm characteristic of APDS (see Fig. 1(a)). As can be clearly seen from Fig. 1(b), upon addition of the Ag sol, the absorption peak at 257 nm gradually decreases and becomes smoother. This overall spectral transition occurs due to the formation of a new compound by the interaction between APDS and the Ag sol. When the amount of Ag sol is small, the APDS in the solution coexists with the newly formed compound. With increasing Ag sol concentration, as more of the new compound forms and less APDS is present in solution the absorption peak at 257 nm is diminished. Concurrent with the gradual weakening of the absorption peak located at 257 nm with Ag sol addition, a new absorption peak at 970 nm gradually appears. And with increasing the concentration of Ag-sol, the absorption peaks increase continually. This new absorption peak corresponds to the presence of the new compound formed by the reaction of APDS with the Ag sol. The absorption peak at 450 nm corresponds to the silver nanoparticles. Similarly, upon increasing its amount, the intensity of the peak at 450 nm also gradually increases, even though whether or not there is aggregation of the nanoparticles. Generally speaking, the plasma-assisted coupling reaction can be indicated from UV-vis spectra, due the occurring of a new peak at 970 nm. However, it can’t provide the information of the reaction mechanism. So Raman spectra are need for investigating the detailed reaction information.

**Figure 2:**
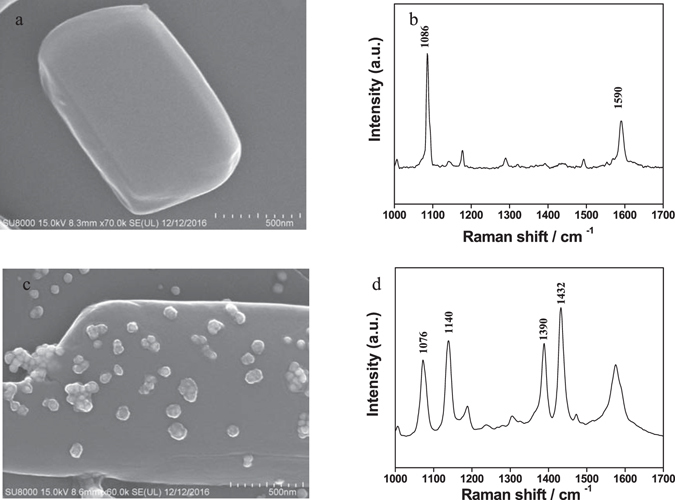
SEM images and Raman spectra of pure APDS and APDS covered with the silver nanoparticles. (**a**) The SEM image of pure APDS; (**b**) the Raman spectrum of pure APDS under laser excitation at 633 nm; (**c**) the SEM image of APDS covered with silver nanoparticles and (**d**) the SERS spectrum of APDS covered with silver nanoparticles under laser excitation at 633 nm.

A small amount of APDS powder was placed on a clean glass slide and then gently pressed with another clean slide, allowing a smooth interface between APDS and the surface of the slide. A mixture of Ag sol and ethanol (1:1 v/v) were dropped onto the glass slide with APDS on the surface and allowed to dry in air. Figure 2(a) shows a SEM image of pure APDS and Fig. 2(b) shows the Raman spectrum of pure APDS under laser excitation at 633 nm. Figure 2(c) shows the SEM image of APDS covered with silver nanoparticles and Fig. 2(d) the Raman spectrum (SERS) of APDS covered with Ag NPs under laser excitation at 633 nm. It can be seen from Fig. 2(c) that the silver nanoparticles are uniformly distributed on the surface of APDS and the particle size of the silver nanoparticles is 50–90 nm. By comparing Fig. 2(b) and (d), it can be seen that the overall Raman spectra differ significantly. In particular, Fig. 2(b) shows the spectrum of pure APDS with characteristic peaks at 1086 and 1590 cm^−1^, assigned to the phenylene-S stretching vibrations and benzene ring vibrations, respectively. Contrasting this, Fig. 2(d) shows three new intense Raman peaks at 1140, 1390 and 1432 cm^−1^. These new peaks were previously considered to be representative peaks of the SERS enhancement effect, but were later identified by Sun *et al*. as the characteristic peak of a new substance, DMAB^8, 9^, which form via a surface plasma assisted reaction. The peak at 1140 cm^−1^ corresponds to the C-N vibrational mode and the peaks at 1390 and 1432 cm^−1^ correspond to the -N=N- vibrational modes^8, 23, 24^. The three Raman peaks in Fig. 2(d) show that when the silver nanoparticles are present on the surface of the APDS, DMAB is generated due to the plasma-assisted catalytic reaction of APDS under laser irradiation.

**Figure 3:**
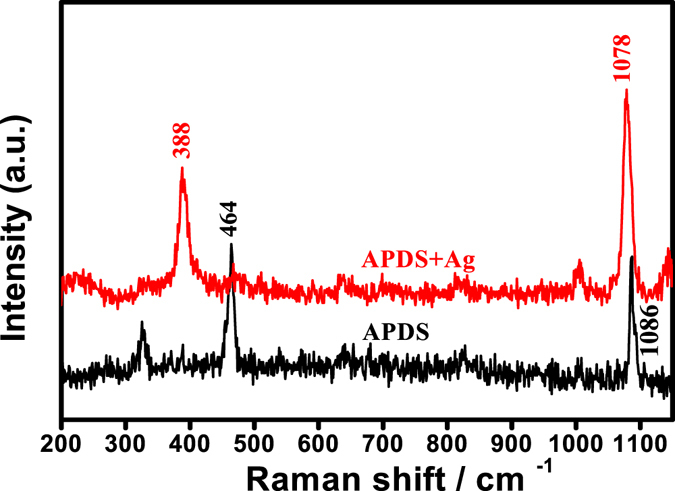
Raman and SERS spectra of pure APDS and APDS covered with silver nanoparticles irradiated using a 785 nm laser at 0.00005% power.

To further verify that the laser induced plasma assisted APDS chemical reaction generates DMAB, we chose a smaller energy wavelength laser (785 nm) as the excitation light source (power = 1.5 × 10^−4^ mW). Figure 3(a) and (b) correspond to the NRS and SERS spectra of DAPS under laser excitation at 785 nm, respectively. From this it can be seen that when the 785 nm excitation light source was utilized on the surface of APDS with small silver nanoparticles, the Raman peak located at 464 cm^−1^, which was attributed to the S-S stretching vibration disappears and a new Raman peak appears at 388 cm^−1^ ^2^. This observation clearly shows the disappearance of the S-S bond due to S-S bond cleavage, and concomitant formation of Ag-S bonds on the surface of APDS^25^. Parallel with this, the characteristic peak at 1086 cm^−1^, which was attributed to the phenylene-S stretching vibration mode in the NRS spectrum of APDS, is shifted to 1078 cm^−1^ in the SERS spectrum. This observation also clearly shows that the S-S bond in APDS breaks and forms an S-Ag bond with the silver nanoparticles^25, 26^.

The enhancement peak of APDS itself was observed at low power (0.00005%) and not the characteristic peak of DMAB, which indicates that in the case of weak laser excitation (low plasma concentration) of APDS only SERS enhancement occurs and no chemical change. This indicates that the presence of plasma, and in high concentration, is necessary for the conversion of APDS to DMAB, i.e., this reaction of APDS to DMAB is catalysed by plasma.

**Figure 4:**
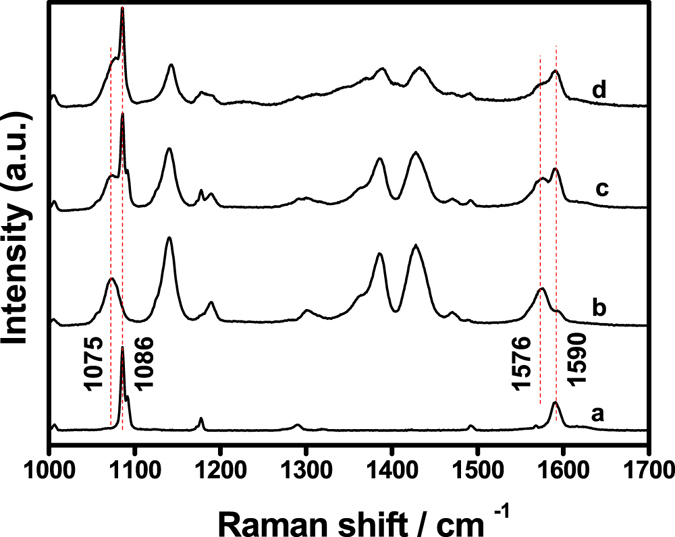
Raman spectra of pure APDS and DMAB generated under laser excitation at 633 and 785 nm. (**a**) The spectrum of pure APDS; (**b**) the spectrum of DMAB generated from APDS under laser excitation at 633 nm; (**c**) the spectrum obtained by combining the spectra of (**a**) and (**b**) calculated using wire 4.2 software provided by Renishaw (United Kingdom) and (**d**) the SERS spectrum of APDS obtained upon laser excitation at 785 nm.

Figure 4 shows the Raman spectra of pure APDS, and DMAB generated under laser excitation at 633 and 785 nm. Figure 4(a) is the Raman spectrum of pure APDS and Fig. 4(b) is the spectrum of DMAB generated from APDS under laser excitation at 633 nm. Figure 4(c) is the simple algebraic superposition spectrum obtained by combining the spectra of Fig. 4(a) and (b) using wire 4.2 software provided by Renishaw (United Kingdom). Figure 4(d) shows the SERS spectra of APDS obtained upon laser excitation at 785 nm. It can be seen from Fig. 4(d) that the SERS spectra of APDS shows a significant change when compared with the NRS spectra when laser excitation power is increased to 5% under laser excitation at 785 nm. In particular, three distinctive Raman peaks appear at 1140, 1390, and 1432 cm^−1^. This indicates that under laser excitation at 785 nm, when the laser power is increased, APDS can also be converted to DMAB in the presence of the plasma-assisted catalyst. However, as can be seen from Fig. 4(c) and (d), there is a significant difference in the curves of DMAB obtained upon laser irradiation at 785 and 633 nm. In Fig. 4(d), a sharp peak appears at 1086 cm^−1^ and the Raman peak at 1075 cm^−1^ of the original DMAB excited at 633 nm becomes a shoulder. Alongside this, the Raman spectra of the DMAB excited at 785 nm shows an enhanced peak at 1590 cm^−1^, resulting in a double peak at 1576 cm^−1^. This is significantly different from the DMAB curve obtained upon laser excitation at 633 nm (see Fig. 4(b)). From the comparison between the Raman spectrum of pure APDS (Fig. 4(a)) with the SERS spectrum excited by laser irradiation at 785 nm (Fig. 4(d)), it can be seen that the sharp peak at 1086 cm^−1^ and the enhancement peak at 1590 cm^−1^ were also found in the NRS of pure APDS. It is therefore likely that the resulting SERS spectrum excited using laser irradiation at 785 nm is the spectrum of a mixture of APDS and DMAB. By further comparing Fig. 4(c) with 4(d), it can be seen that they are very similar. In particular, the sharp peak at 1086 cm^−1^, the shoulder peak at 1075 cm^−1^, the enhanced peak at 1590 cm^−1^ and the double peak at 1576 cm^−1^. Hence, Fig. 4 clearly supports our rationalisation that laser excitation at 785 nm results in a mixture of APDS and DMAB.

The Raman spectrum under laser excitation at 785 nm is the spectrum of the mixture due to the exposed range of 785 nm laser is a line source, instead of a point light source as 633 nm laser. As the excitation light source in the measured sample uses a spot length of 10 microns, this long spot range it is difficult to be covered by silver nanoparticles. Therefore, APDS coated with silver nanoparticles and APDS uncovered with silver nanoparticles are together exposed in the 785 nm laser irradiated range. When laser irradiation of the silver nanoparticles on the surface occurs, a large amount of plasma is produced. Under the conditions of this plasma-assisted catalysis, APD was converted to DMAB and the characteristic spectrum of DMAB was obtained. When the irradiation was applied to a pure APDS surface, not covered by silver nanoparticles, the characteristic spectrum of pure APDS was obtained as no plasma is generated. Therefore, laser excitation at 785 nm results a mixture of APDS and DMAB. This proves that the conversion of APDS to DMAB can only occur when the silver nanoparticles are present at a sufficient concentration.

**Figure 5:**
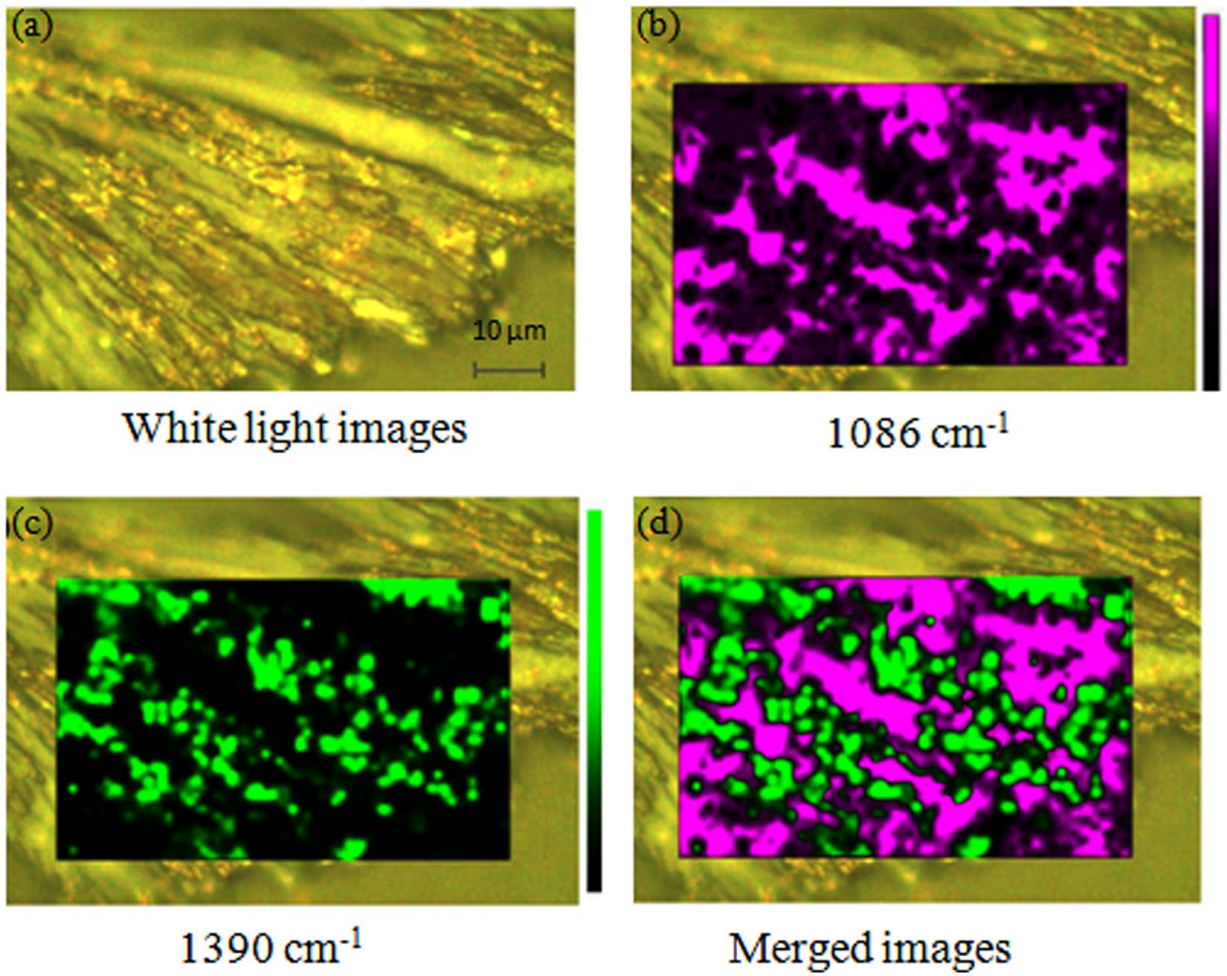
Images of APDS partially covered with silver nanoparticles. (**a**) White light images, (**b**) mapping image of the peak position at 1086 cm^−1^ using wire 4.0 software and (**c**) mapping image of the peak at 1390 cm^−1^ using wire 4.0 software, (**d**) merged images of (**b**) and (**c**).

In order to verify this theory we conducted an in-depth experiment whereby a specific region covered by the silver nanoparticles was selected and mapped using the 633 nm laser to obtain the related images. Figure 5 shows the optical image and Raman mapping image of APDS partially covered with silver nanoparticles. Figure 5(a) is the white light image, Fig. 5(b) and (c) are the mapping image of the peak position at 1086 cm^−1^ and 1390 cm^−1^ using wire 4.0 software, Fig. 5(d) is the merged image of Fig. 5(c) and (d). It can be seen from Fig. 5(a) that, since APDS was dissolved and dried in ethanol, there are obvious flow traces which are distributed like a mountain-like radiator. Owing to the flow of ethanol, the silver nanoparticles in the valley are washed away by ethanol and the silver nanoparticles left in the ridges emit a metallic lustre under irradiation with white light.

Raman scanning imaging was performed on the selected region. Figure 5(b) is the mapping image of the peak at 1086 cm^−1^ (using wire 4.0 software), where the green spot represents the position of the Raman peak at 1086 cm^−1^ and the relative intensity of the light represents the relative intensity of the Raman peak at 1086 cm^−1^. As can be seen from Fig. 2, the sharp peak appears at 1086 cm^−1^ in the Raman spectrum of pure APDS, relative to the spectrum of DMAB. Therefore, we believe that at the physical location of the peak at 1086 cm^−1^, there are no silver nanoparticles in the laser spot. Thus, the pattern of Fig. 5(b), represented by a pink spot, represents the distribution of pure APDS. Figure 5(c) is the mapping image of the peak at 1390 cm^−1^ (using wire 4.0 the software). As mentioned previously, the peak at 1390 cm^−1^ can be attributed to Raman vibrations generated by N=N vibrational modes, so the green spot in Fig. 5(c) represents the DMAB distribution due to plasma assisted catalysis. By comparing Fig. 5(a) and (c), the green spots show the distribution of the metallic lustre of the silver nanoparticles position which is almost equivalent. This confirms the accuracy of our labelling of DMAB at 1390 cm^−1^ in the Raman spectra, i.e., where laser irradiation occurs on the APDS surface where silver nanoparticles are present and where plasma assisted catalysis (APDS) generates DMAB. Figure 5(d) is the merged image of Fig. 5(b) and (c). As can be seen more clearly in Fig. 5(d), the green and pink spots almost cover the entire experimental area and the green spots are radially distributed, which is consistent with the distribution of the nanoparticles shown in Fig. 5(a). This means, under the irradiation of the laser, the APDS coated with silver nanoparticles produces DMAB under plasma-assisted catalysis and shows the characteristic peak of DMAB in the Raman spectrum.

Overall, the results we have obtained from a wide range of scanning experiments are in full accordance with previously reported theories. This study has also shown that spectroscopic monitoring of the plasma catalytic reaction is very accurate and has a high recognition and repetition rate.

**Figure 6:**
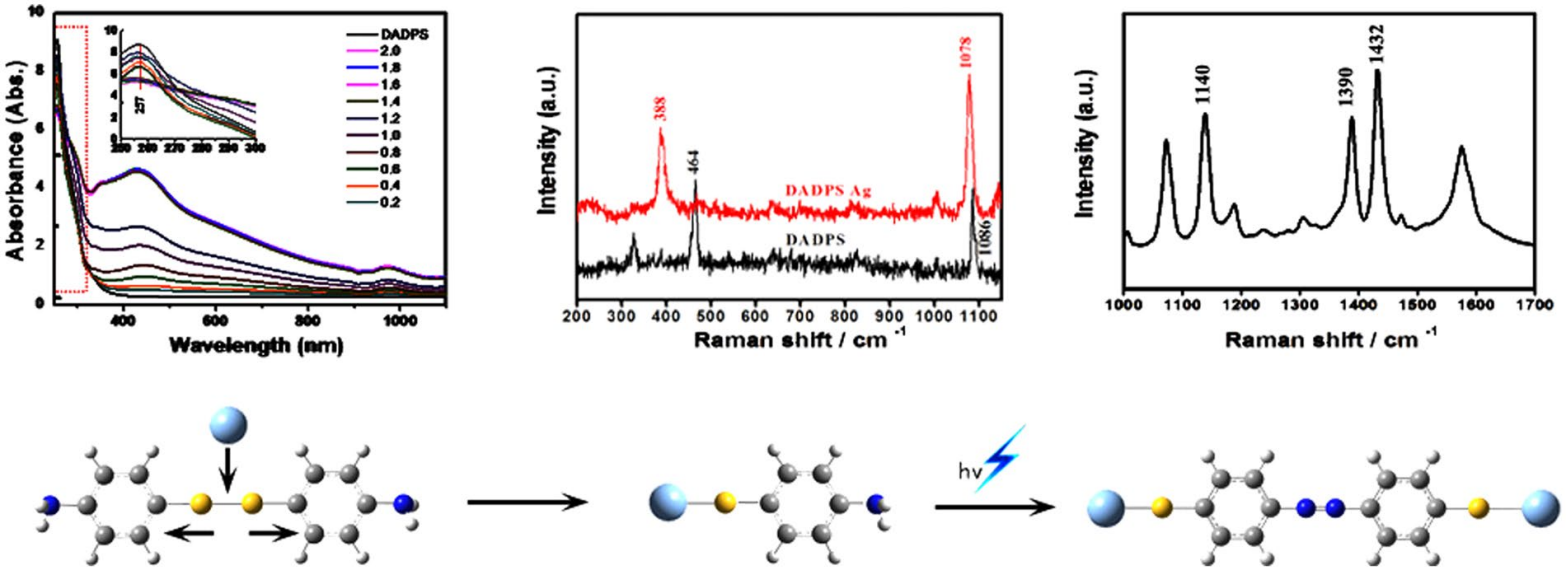
The spectroscopy-proven process of the APDS plasma-assisted catalytic reaction.

The spectrum monitoring process of DMAB generated from APDS in the plasma assisted photo-catalytic reaction is shown in Fig. 6. Firstly, when the APDS is in contact with the silver nanoparticles the S-S bonds break resulting in the formation of a new material. This process is reflected in the ultraviolet spectrum of 257 and 1000 nm near the absorption peak. Secondly, the Raman peaks at 464 cm^−1^ disappear and a new peak appears at 388 cm^−1^ when APDS is excited by the low energy laser (785 nm). This indicates that the S-S bonds in APDS break to form a new compound (NH_2_-C_6_H_6_-S-Ag) via S-Ag bond formation with the silver nanoparticles. Finally, the characteristic peaks at 1140, 1390 and 1432 cm^−1^ can be clearly observed by Raman spectroscopy. This indicates that two NH_2_-C_6_H_6_-S-Ag units form a new compound, DMAB, through the formation of N=N double bonds under plasma-assisted catalysis.

## Conclusions

In summary, we provided the experimental evidence for detecting the plasma-assisted coupling reaction from APDS to DMAB. Our results indicate that when the 633 nm laser was selected as the excitation source, DMAB was quickly generated following mixing PATP with Ag nanoparticles. But this process is too fast to be well detected by Raman spectroscopy. For further clearly indentifying the mechanism of DMAB generation from APDS by SERS effect, a light source with lower energy (785 nm laser) was chosen as the excitation source. The results show that DMAB is formed through the S-S cleavage and Ag-S formation. This technology has a high degree of recognition and a high repetition rate, and has potential applications in biosensors, bio-fast imaging of living cells and other fields.

## Methods

Silver nitrate (>99.95%), trisodium citrate (99%), APDS and absolute ethanol were purchased from commercial suppliers and all aqueous solutions were prepared using 18 MΩ high purity water. Nanosize silver particles were obtained via the citric acid reduction of silver nitrate solution (1.00 mmol L^−1^). The silver nitrate solution was heated at reflux, 10 mL of sodium citrate solution (1 wt%) was added and the resulting solution kept at reflux for 1 h with stirring and then allowed to cool to room temperature.

Placing a small amount of APDS onto a clean slide, the APDS was gently pressed onto the slide’s surface using another clean slide to smoothen the sample’s surface. Ag sol and ethanol 1:1 (v/v) were mixed and then dropped on the glass slides with APDS and allowed to dry in air whilst measuring the Raman spectra, ultraviolet spectra and scanning electron microscopy (SEM).

All the Raman spectra of each sample were recorded using Renishaw inVia and the absorption spectra were recorded using Optizen 2120UV.

## References

[1] Berger AG, Restaino SM, White IM (2017). Vertical-flow Paper SERS System for Therapeutic Drug Monitoring of Flucytosine in Serum. Anal. Chim. Acta.

[2] Moskovits M (1985). Surface-enhanced Spectroscopy. Rev. Mod. Phys..

[3] Sun MT (2013). Remotely excited Raman optical activity using chiral plasmon propagation in Ag nanowires. Light Sci. Appl.

[4] Tian ZQ, Bin Ren A, Wu DY (2002). Surface-Enhanced Raman Scattering:  From Noble to Transition Metals and from Rough Surfaces to Ordered Nanostructures. J. Phys. Chem. B.

[5] Zheng H, Ni D, Yu Z, Liang P (2017). Preparation of SERS-Active Substrates Based on Graphene Oxide/silver Nanocomposites for Rapid Zdetection of l-Theanine. Food Chem..

[6] Henry AI, Sharma B, Cardinal MF, Kurouski D, Duyne RPV (2016). SERS Biosensing: *in vivo* Diagnostics and Multimodal Imaging. Anal. Chem..

[7] Ni, Z. H. *et al*. Raman Spectroscopy of Epitaxial Graphene on a SiC Substrate. *Phys. Rev. B* **77** (2008).

[8] Fang Y, Li Y, Xu H, Sun M (2010). Ascertaining p, p′-Dimercaptoazobenzene Produced from p-Aminothiophenol by Selective Catalytic Coupling Reaction on Silver Nanoparticles. Langmuir.

[9] Huang Y, Fang Y, Yang Z, Sun M (2010). Can p,p′-Dimercaptoazobisbenzene Be Produced from p-Aminothiophenol by Surface Photochemistry Reaction in the Junctions of a Ag Nanoparticle–Molecule–Ag (or Au) Film?. J. Phys. Chem. C.

[10] Sun M, Huang Y, Xia L, Chen X, Xu H (2011). The pH-Controlled Plasmon-Assisted Surface Photocatalysis Reaction of 4-Aminothiophenol to p,p′-Dimercaptoazobenzene on Au, Ag, and Cu Colloids. J. Phys. Chem. C.

[11] Sun M, Hou Y, Li Z, Liu L, Xu H (2011). Remote Excitation Polarization-Dependent Surface Photochemical Reaction by Plasmonic Waveguide. Plasmonics.

[12] Dong B, Fang Y, Chen X, Xu H, Sun M (2011). Substrate-, Wavelength-, and Time-Dependent Plasmon-Assisted Surface Catalysis Reaction of 4-Nitrobenzenethiol Dimerizing to p,p′-Dimercaptoazobenzene on Au, Ag, and Cu Films. Langmuir.

[13] Sun M, Zhang Z, Zheng H, Xu H (2012). *In-Situ* Plasmon-driven Chemical Reactions Revealed by High Vacuum Tip-Enhanced Raman Spectroscopy. Sci. Rep.-UK.

[14] Zhang Z (2013). Insights into the Nature of Plasmon-Driven Catalytic Reactions Revealed by HV-TERS. Nanoscale.

[15] Xu P (2013). Mechanistic Understanding of Surface Plasmon Assisted Catalysis on a Single Particle: Cyclic Redox of 4-Aminothiophenol. Sci. Rep.-UK.

[16] Zhang X, Wang P, Zhang Z, Fang Y, Sun M (2014). Plasmon-Driven Sequential Chemical Reactions in an Aqueous Environment. Sci. Rep.-UK.

[17] Zhang Z, Xu P, Yang X, Liang W, Sun M (2016). Surface Plasmon-Driven Photocatalysis in Ambient, Aqueous and High-Vacuum Monitored by SERS and TERS. J. PhotochPhoto C.

[18] Cui, L., Wang, P., Li, Y. & Sun, M. Selective Plasmon-Driven Catalysis for para-Nitroaniline in Aqueous Environments. *Sci. Rep.-UK* **6** (2016). 10.1038/srep20458 PMC4746588 26857259

[19] Cui L (2016). Plasmon-Driven Catalysis in Aqueous Solutions Probed by SERS Spectroscopy. J. Raman Spectrosc..

[20] Kang L (2013). Laser Wavelength- and Power-Dependent Plasmon-Driven Chemical Reactions Monitored Using Single Particle Surface Enhanced Raman Spectroscopy. Chem. Comm.

[21] Azcune I, Odriozola I (2016). Aromatic Disulfide Crosslinks in Polymer Systems: Self-healing, Reprocessability, Recyclability and More. Eur. Polym. J..

[22] Luzuriaga ARD (2016). Transient Mechanochromism in Epoxy Vitrimer Composites Containing Aromatic Disulfide Crosslinks. J. Mater. Chem. C.

[23] Huang YF (2010). When the Signal is not from the Original Molecule to be Detected: Chemical Transformation of para-Aminothiophenol on Ag During the SERS Measurement. J. Am. Chem. Soc..

[24] Dong B, Fang Y, Xia L, Xu H, Sun M (2011). Is 4-Nitrobenzenethiol Converted to p,p ‘-Dimercaptoazobenzene or 4-Aminothiophenol by Surface Photochemistry Reaction?. J. Raman Spectrosc..

[25] Li F, Lu Y, Ren X (1996). The FT-Raman Study of Reaction of 4-Aminophenyl Disulfide and Epoxy on Silver Surface. Spectrosc. Lett..

[26] Dai Q, Xue C, Xue G, Jiang L (1995). Fourier-Transform Surface Enhanced Raman Scattering Studies of Molecular Aelf-Assembly of Disulfides on Metals and the Application in Adhesion Promotion. J. Adhes. Sci. Technol..

